# The modulation of temporal predictability on attentional boost effect

**DOI:** 10.1002/brb3.3653

**Published:** 2024-09-01

**Authors:** Jianan Pan, Chao Fu, Ping Su, Qian Guo, Xinglin Li, Chun Zheng, Xueqin Ma, Tingjun Yong

**Affiliations:** ^1^ Department of Psychology School of Education Qinghai Normal University Xining Qinghai P.R. China; ^2^ Academy of Plateau Science and Sustainability People's Government of Qinghai Province & Beijing Normal University Xining Qinghai P.R. China; ^3^ Plateau Medical Research Center Xining Qinghai China; ^4^ Qinghai Cardio‐Cerebrovascular Specialty Hospital Qinghai High Altitude Medical Research Institute Xining Qinghai P.R. China; ^5^ Department of Psychology School of Education Qinghai Minzu University Xining Qinghai P.R. China; ^6^ Department of Gastroenterology Cardio‐cerebrovascular Disease Hospital Xining Qinghai P.R. China; ^7^ School of Education Shaanxi Normal University Xi'an Shaanxi P.R. China

**Keywords:** attentional boost effect, behaviorally relevant events, perceptual enhancement, predictability, temporal orienting

## Abstract

**Introduction:**

The attentional boost effect, characterized by better memory for background scenes coinciding with a detection target than a nontarget, is believed to stem from a temporary increase in attentional capacity at the time of an acute behavior‐related event occurring. Sisk and Jiang's study found that the attentional boost effect also occurs when the target's appearance was predictable. Unfortunately, the duration of the predictive interval in Sisk and Jiang's study was fixed. Since different predictive intervals had different weakening degrees to the acuteness of the target, this fixed duration hindered further investigation into the impact of different levels of predictability on the attentional boost effect.

**Method:**

Using the encoding‐recognition paradigm and the remembering/knowing paradigm, and setting target stimuli with different predictive interval in target detection tasks, the current study aimed to explore the influence of varying the duration of the predictive interval on the attentional boost effect.

**Results:**

The attentional boost effect was observed only in the short and medium predictive duration conditions, but not in the long predictive duration condition. Moreover, as the duration of the predictive interval increased, participants’ memory performance on target‐paired words gradually declined, while their memory performance on distractor‐paired and baseline‐paired words gradually improved.

**Conclusions:**

Predictability may alter the task demands, allowing participants to more effectively allocate attentional resources to the two tasks at hand.

## Graphical abstract

This study investigates the attentional boost effect (ABE) and its relationship with event predictability. Using encoding‐recognition and remembering/knowing paradigms, it was found that short and medium predictive intervals enhance memory for target‐paired words, while long intervals do not. Predictability alters task demands, allowing better allocation of attentional resources. Under short predictability, more attention is given to target stimuli, enhancing memory (ABE). Under long predictability, attention is distributed more broadly, reducing the ABE.

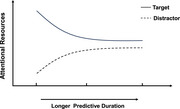

## 1 INTRODUCTION

The relationship between attention and memory remains a focal point in cognitive psychology (Backer & Alain, 2014; Chun & Turk‐Browne, 2007). The resource limitation theory posits that attentional resources are finite (Kahneman, 1973). According to this theory, when multiple tasks are performed simultaneously (divided attention), memory performance may decline due to the competition for limited attentional resources (Kinchla, 1992). Contrary to the resource limitation theory, in their study of long‐term memory, Swallow and Jiang (2010) found that simultaneous completion of a memory task and a target detection task led to improved memory performance, specifically for the material presented concurrently with the target stimulus. This finding challenges the notion that multitasking invariably impairs memory, suggesting that, under certain conditions, “dual‐mindedness” can enhance memory, Swallow and Jiang (2010) term this phenomenon the attentional boost effect (ABE). Subsequent research extended the ABE's applicability to various memory tasks, such as short‐term memory (Makovski et al., 2011), implicit memory (Meng et al., 2021; Spataro et al., 2013), and source memory (Mulligan et al., 2016, 2021). Moreover, the ABE was observed across diverse memory materials, including face memory (Swallow & Jiang, 2012) and verbal memory (Mulligan et al., 2014). This body of evidence underscores the stability of the ABE as a cognitive phenomenon.

To elucidate the ABE, Swallow and Jiang introduced the dual‐task interaction model (Swallow & Jiang, 2013; Swallow et al., 2022). According to this model, the response to an acute target (behaviorally relevant events) triggers temporal selective attention, also known as temporal orienting of attention. This refers to the ability to concentrate resources at a specific moment in time to optimize behavior (Coull et al., 2000). Prior research has indicated that temporal selective attention is often accompanied by the transient electro‐discharge of the locus coeruleus and the release of a substantial amount of norepinephrine into sensory regions of the cerebral cortex. This process leads to a transient but widespread perceptual enhancement, resulting in a temporary increase in attentional capacity (Aston‐Jones & Cohen, 2005; Yebra et al., 2019). It is this transient yet widespread perceptual enhancement that facilitates the encoding and processing of memory materials presented simultaneously with the target event. In short, the emergence of the ABE follows such a logical chain: behaviorally relevant acute events prompt temporal selective attention, which, in turn, triggers perceptual enhancement. This enhanced perceptual state facilitates the encoding process, ultimately resulting in the manifestation of the ABE—better memory for background scenes coinciding with a detection target than a nontarget.

Target stimuli were typically designed to be unpredictive in early studies (Lin et al., 2010; Mulligan et al., 2014; Smith & Mulligan, 2018). However, relevant events need not be unpredictive. Consider driving: The detection of a red traffic light is a highly relevant event requiring a response by the driver. Yet the moment when a light turns red is not unpredictive—the duration of the green light provides some information, and the onset of the yellow light predicts when the red light will appear (Sisk & Jiang, 2020). Thus, one question worth exploring is that whether a predictive target also trigger a temporal orienting response, and in turn, causing a transient attentional boost? To explore this issue, Sisk and Jiang asked participants to monitor a stream of digits for the occurrence of the digit 0 (target digit). In some trial blocks, the occurrence of 0 was unpredictive. In other trial blocks, the countdown sequence 3, 2, 1 always led to 0, and participants were informed of the predictability. Photographs of scenes were presented in the background, and participants were instructed to memorize the scene images. A recognition test was administered later to determine whether scenes coinciding with the target digit were remembered better than scenes coinciding with the nontarget digits. The results showed that the ABE still occurred when the target was predictive (Sisk & Jiang, 2020).

Regrettably, the duration of the predictive interval in Sisk and Jiang's study was fixed (3 s), since the ABE was thought to be caused by a temporary increase of attentional capacity (perceptual enhancement) at the time of an acute behavior‐related event occurring (Sisk & Jiang, 2020), while different predictive intervals (predictability) had different weakening degrees to the acuteness of the target. The use of a fixed duration of the predictive interval thus hindered further investigation into the impact of different levels of predictability on the ABE. Building upon Sisk and Jiang's paradigm (Sisk & Jiang, 2020) and setting target stimuli of different durations of the predictive interval (see Section 2; SPD: short predictive duration; MPD: medium predictive duration; LPD: long predictive duration) in the target detection task, the current study aimed to explore the following issues: (1) target versus distractors: the differences in memory task performance between words coinciding with target digit and words coinciding with nontarget digits (distractive digit); (2) predictive versus unpredictive distractors: the differences in memory task performance between words coinciding with predictive nontarget digits (predictive distractive digit) and words coinciding with unpredictive nontarget digits (unpredictive distractive digit); and (3) distal predictive distractors versus proximal predictive distractors: the differences in memory task performance between words coinciding with distal predictive nontarget digits and words coinciding with proximal predictive nontarget digits. As the ABE was thought to be caused by a temporary perceptual enhancement at the time of an acute behavior‐related target event occurring (Sisk & Jiang, 2020), we expected that longer durations of the predictive interval should weaken the acuteness of the target event. Concerning issue 1, we inferred that the longer the duration of the predictive interval, the weaker the ABE would be, and there should be a critical point of the duration of the predictive interval that produces the ABE. Furthermore, according to the Sisk and Jiang, the predictive digits reliably cue the target event, they may also be categorized as behaviorally relevant moments and induce a temporal orienting response. Thus regarding the nontarget distractor digits (issue 2), we hypothesized that the words coinciding with predictive nontarget digits will be better remembered than the words coinciding with unpredictive nontarget digits. Finally, the results of Sisk and Jiang's study showed a small but significant memory advantage for scenes coinciding with the middle digit 2, but not the bilateral digit 1 or 3. For the predictive nontarget digits (issue 3), we thus infer that such an “intermediate position memory advantages” would also appear in our study. Specifically, words coinciding with middle predictive nontarget digits (MPD: 2; LPD: 3, 4) would be better remembered than words coinciding with bilateral predictive nontarget digits (MPD: 1, 3; LPD: 1, 2, 5, 6).

## 2 MATERIALS AND METHODS

### 2.1 Participants

Sample size was guided by previous studies on the attentional boost effect, with typical sample sizes ranging from 12 to 20 participants (Sisk & Jiang, 2020). Based on the effect size of *η_p_* ^2^ = .64 (Sisk & Jiang, 2020; Swallow & Atir, 2018; Swallow & Jiang, 2010, 2013, 2014), G‐power analysis suggests that a minimum of 17 participants was needed to reach a power of 0.80 with an alpha level of 0.05. To ensure a balanced distribution of the three experimental conditions among participants, 24 undergraduates (age: mean ± SD = 18.7 ± 0.46 years) were initially recruited. All participants were right‐handed (according to the Edinburgh Handedness Inventory) (Oldfield, 1971), had normal or corrected‐to‐normal vision, and reported no history of neurological or psychiatric conditions. All participants provided written informed consent according to protocols approved by the local ethics committee. The research protocol was approved by the local Ethics Committee and was in compliance with ethical standards of the American Psychological Association. Written informed consent was obtained from all the participants prior to the enrollment of this study. Each participant received a small monetary remuneration.

### 2.2 Materials

In this study, we selected a set of 990 two‐character, low‐frequency, emotionally neutral Chinese words from the Modern Chinese Frequency Dictionary (1986) as our experimental materials. These words were then randomly divided into six sets, each containing 165 words. Three sets (165 × 3 = 495 words) were assigned for the encoding phase, while another three sets (165 × 3 = 495 words) were presented as new words during the recognition phase.

### 2.3 Design and procedure

To better understand the properties of ABE under predictive conditions, we incorporated a baseline condition into the detection task, as suggested by previous studies (Swallow & Jiang, 2014). In this baseline condition, only the word without the number was presented. Since only the memory task was included in this condition, it was also referred to as the focused attention condition, contrasting with the divided attention condition, which included both the memory task and the target detection task. Specifically, the present study employed a 3 × 3 within‐subjects design. The duration of the predictive interval was manipulated across three levels: short predictive duration (SPD), medium predictive duration (MPD), and long predictive duration (LPD). Additionally, the detection stimuli type included three levels: target stimuli (target digit 0), distractor stimuli (nontarget digits: 1–9), and baseline stimuli (only the word and without the digit were presented). Each predictive duration condition consisted of two phases: an encoding phase and a recognition phase. To mitigate order effects, the sequence of the three predictive duration conditions was counterbalanced across participants. The entire experiment was completed within approximately 30 min.

#### 2.3.1 Encoding phase

**FIGURE 1:**
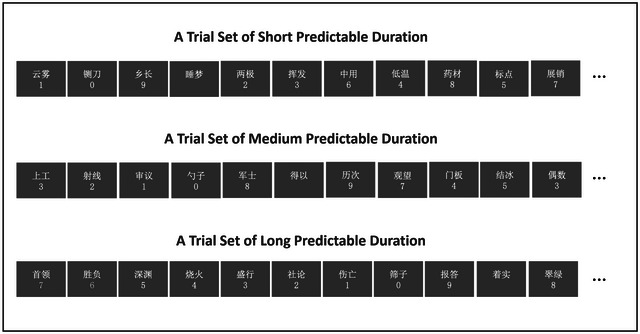
Flow chart of encoding phase. Using the MPD condition as an example, unpredictive nontarget digits 4, 5, 6, 7, 8, and 9 were randomly inserted between the number strings (3‐2‐1‐0). Here, 0 served as the target stimulus, and the digits preceding it (3, 2, and 1) acted as the predictive duration cues. Both the predictive nontarget digits (3, 2, 1) and the randomly inserted unpredictive nontarget digits (4, 5, 6, 7, 8, 9) served as distractor stimuli. Trials featuring only words without numbers were considered baseline stimuli, and these baseline stimuli were also randomly inserted between the number strings. Fifteen trials were included in each predictive duration condition, resulting in a total of 165 words required. (1 target word + 9 nontarget words + 1 baseline word) × 15 = 165. Thus, across the three predictive duration conditions, a total of 495 words (165 words × 3 predictive duration conditions) were needed for the entire encoding phase.

Participants were asked to perform a target detection task while simultaneously memorizing a list of randomly presented words at the center of the screen (Figure 1). In the SPD condition, countdown number strings 1‐0 (target digit: 0, predictive nontarget digit: 1), randomly inserted baseline word, and unpredictive nontarget digits: 2, 3, 4, 5, 6, 7, 8, 9 appeared sequentially. For the MPD condition, countdown number strings 3‐2‐1‐0 (target digit: 0, predictive nontarget digits: 3, 2, 1), randomly inserted baseline word, and unpredictive nontarget digits: 4, 5, 6, 7, 8, 9 appeared sequentially. In the LPD condition, countdown number strings 7‐6‐5‐4‐3‐2‐1‐0 (target digit: 0, predictive nontarget digits: 7, 6, 5, 4, 3, 2, 1), randomly inserted baseline word, and unpredictive nontarget digits: 8, 9 appeared sequentially. Fifteen trials were included in each predictive duration condition, resulting in a total of 165 words required. (1 target word + 9 nontarget words + 1 baseline word) × 15 = 165. Thus, across the three predictive duration conditions, a total of 495 words (165 words × 3 predictive duration conditions) were needed for the entire encoding phase.

Participants were instructed to press the spacebar when the target digit 0 appeared. Digits in front of 0 in the number string (predictive nontarget digits; SPD: 1; MPD: 3, 2, 1; LPD: 7, 6, 5, 4, 3, 2, 1) and randomly inserted digits between the number strings (unpredictive nontarget digits; SPD: 2, 3, 4, 5, 6, 7, 8, 9; MPD: 4, 5, 6, 7, 8, 9; LPD: 8, 9) all served as distractor stimuli. Participants did not need to respond to distractor stimuli and baseline stimuli. Without differentiating between predictive and unpredictive distractors, the target‐to‐distractor ratio remained consistent at 1:9 across all three conditions. For distractors, in SPD, MPD, and LPD conditions, the ratio of predictive to unpredictive distractors (nontarget digit) was 1:8, 3:6, and 7:2, respectively. Across all predictive duration conditions, the digit consistently appeared 1 cm below the word. The digit vanished after a simultaneous 150 ms presentation with the word, followed by the continuous display of the word for 350 ms and a subsequent blank screen lasting 500 ms. To acquaint participants with the experimental procedure, 10 practice trials were provided for each predictive duration condition before the formal experiment. The experiment's procedure was programmed using Psychopy3 software, and stimulus material was presented on a 19‐inch color LCD monitor with a resolution of 1920 × 1080.

#### 2.3.2 Recognition phase

**FIGURE 2:**
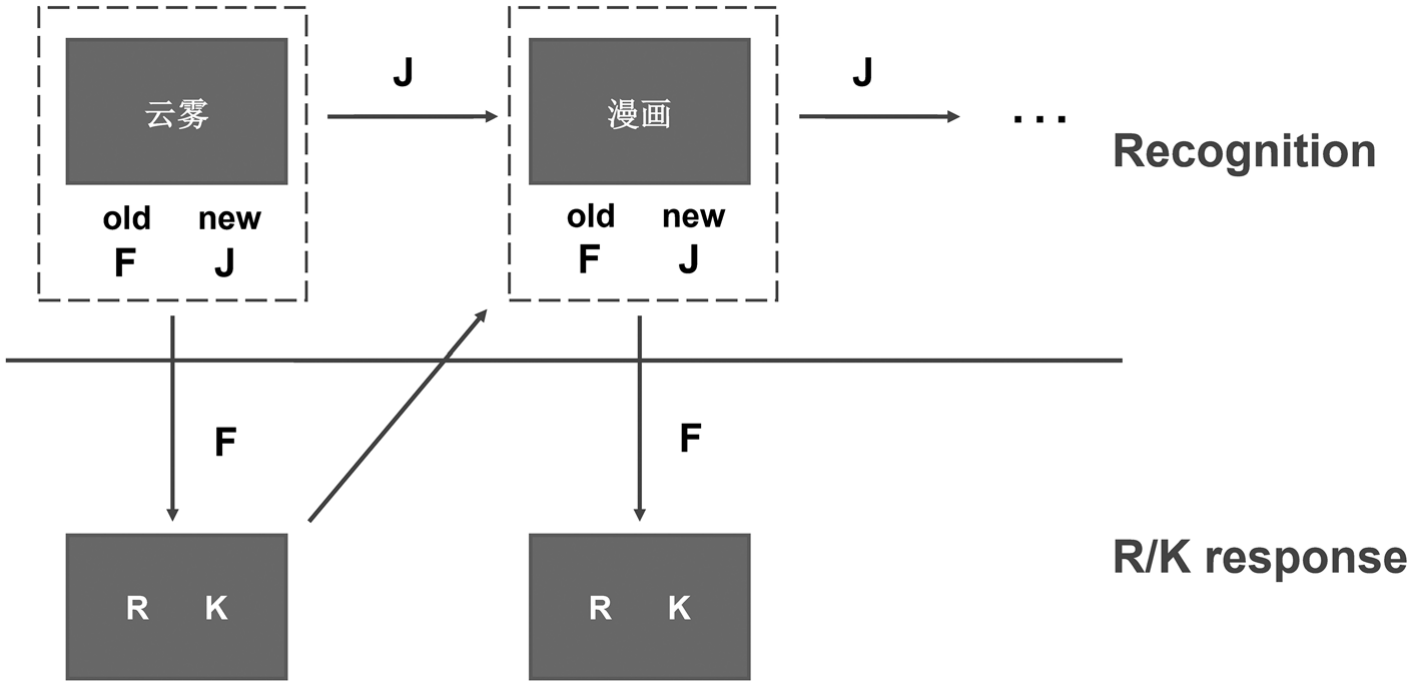
Flow chart of recognition phase. A total of 330 words, comprising 165 old and 165 new words, were randomly presented. Participants were instructed to use the “J” key for new words and the “F” key for old words. After pressing “J,” the next trial began. If “F” was pressed, two additional response options (“remember” and “know,” denoted as R/K) appeared. Participants were then prompted to decide whether they vividly remembered the word (by pressing “R”) or simply had a sense of knowing (by pressing “K”).

After the encoding phase in each predictive duration condition, participants underwent an untimed old/new recognition test (Figure 2). The test comprised 165 old words and 165 new words, randomly presented on the screen. Participants were instructed to press the “J” key for new words and the “F” key for old words. Upon pressing “J” (new), the next trial commenced. If “F” (old) was pressed, two additional response options, “remember” (R) and “know” (K), appeared. Participants were advised to press “R” if they vividly remembered the word and could recall details, and “K” if they had a general sense of knowing without full certainty (Leclercq et al., 2014; Meng et al., 2019).

### 2.4 Statistical analysis

ANOVA for repeated measurement was mainly used in the current study. For all ANOVA for repeated measurements, descriptive data were presented as arithmetic mean ± standard error. The significance level was set at *p* < .05. Greenhouse‐Geisser correction was used whenever appropriate. All pairwise comparisons used Bonferroni correction. Partial eta‐squared (*η_p_* ^2^) values were provided as the demonstration of effect size.

## 3 RESULTS

### 3.1 The accuracy of target detection task

The statistical results showed that the mean accuracy of the target detection tasks under SPD, MPD, and LPD was 96.3 ± 3.4%, 97.2 ± 2.0%, and 96.8 ± 2.1%, respectively. One‐way ANOVA results indicated no significant difference in mean accuracy among the three predictive duration conditions, *F*(1.32, 30.44) = 1.63, *p* = .214, *η_p_* ^2^ = .07, suggesting that participants effectively completed the target detection tasks across all conditions.

### 3.2 Corrected recognition rates of recognition phase

Hit rates and false alarm rates of memory task was showed in Table 1. Considering the potential influence of false alarm rates on the results, corrected recognition rates were calculated by subtracting false alarm rates from hit rates. This corrected recognition rate was then used as the index during statistical analyses in Section 3.2.

**TABLE 1:** Hit rates and false alarm rates of memory task.

	Target	Distractor	Baseline	FAs
SPD	0.41 ± 0.03	0.21 ± 0.03	0.22 ± 0.03	0.12 ± 0.02
MPD	0.36 ± 0.03	0.29 ± 0.03	0.29 ± 0.04	0.15 ± 0.03
LPD	0.33 ± 0.03	0.29 ± 0.04	0.31 ± 0.04	0.18 ± 0.04

*Note*: Descriptive data were presented as arithmetic mean ± standard error

SPD: short predictive duration; MPD: medium predictive duration; LPD: long predictive duration.

#### 3.2.1 Target versus distractors

**FIGURE 3:**
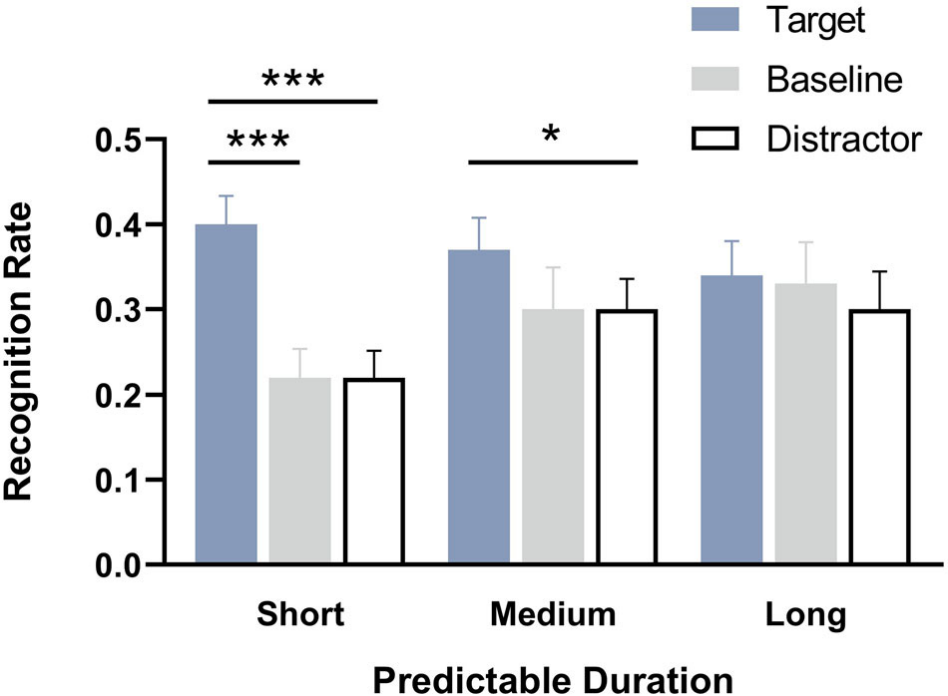
The corrected recognition rates for words paired with different detection stimuli types across the three predictive duration conditions. Error bars represent standard error of the mean; *p < .05, **p < .01, ***p < .001.

A 3 (predictive duration: SPD, MPD and LPD) × 3 (detection stimuli type: target, distractor and baseline) repeated measures ANOVA on corrected recognition rates (Figure 3) indicated that the main effect of predictive duration was not significant, *F*(1.70, 36.70) = 1.32, *p *= .276, *η_p_* ^2^ = .05; the main effect of detection stimuli type was significant, *F*(1.50, 34.51) = 24.78, *p* < .001, *η_p_* ^2^ = .52. Pairwise comparisons revealed that the corrected recognition rates for target‐paired words (0.22 ± 0.02) were significantly higher than those for baseline‐paired words (0.13 ± 0.02, *p* < .001) and distractor‐paired words (0.11 ± 0.01, *p* < .001). No significant differences were found between corrected recognition rates for baseline‐paired words (0.13 ± 0.02) and distractor‐paired words (0.11 ± 0.01, *p* = .619). Above repeated measures ANOVA also revealed a significant interaction between predictive duration and detection stimuli type, *F*(4, 92) = 6.93, *p* < .001, *η_p_* ^2^ = .23. Follow‐up simple effect analyses demonstrated a significant effect of detection stimuli type in the SPD condition, *F*(2, 22) = 27.47, *p* < .001, *η_p_* ^2^ = .71. Specifically, corrected recognition rates for target‐paired words (0.29 ± 0.03) were significantly higher than those for baseline‐paired words (0.11 ± 0.02, *p* < .001) and distractor‐paired words (0.09 ± 0.02, *p* < .001). No significant differences were found between the corrected recognition rates of baseline‐paired words (0.11 ± 0.02) and those of distractor‐paired words (0.09 ± 0.02, *p* = 1). In the MPD condition, the simple effect of detection stimuli type was significant, *F*(2, 22) = 6.38, *p* = .007, *η_p_* ^2^ = .37. Corrected recognition rates for target‐paired words (0.21 ± 0.02) were significantly higher than those for distractor‐paired words (0.14 ± 0.02, *p* = .005). No significant differences were found between corrected recognition rates for target‐paired (0.21 ± 0.02) and baseline‐paired words (0.14 ± 0.03, *p* = .128) and between distractor‐paired (0.14 ± 0.02) and baseline‐paired words (0.14 ± 0.03, *p* = 1). However, the simple effect in the LPD condition was not significant, *F*(2, 22) = 2.47, *p* = .108, *η_p_* ^2^ = .18. As depicted in Figure 3, there is a noticeable trend in the corrected recognition rates of target‐paired words (blue pillar), which exhibit a gradual decrease with increasing predictive duration. Conversely, the corrected recognition rates of baseline‐paired words (gray pillar) and distractor‐paired words (hollow pillar) demonstrate a gradual increase. Consequently, we conducted separate simple effect tests to analyze the influence of different types of detection stimuli words across varying predictive durations. The results showed that for the target‐paired words, the simple effect of predictive duration was significant, *F*(2, 22) = 6.08, *p* = .008, *η_p_* ^2^ = .36. Corrected recognition rates in the SPD condition (0.29 ± 0.03) were significantly higher than in the MPD condition (0.21 ± 0.03, *p *= .050) and LPD condition (0.15 ± 0.03, *p *= .003). Moreover, the corrected recognition rates in the MPD condition (0.21 ± 0.03) were significantly higher than in the LPD condition (0.15 ± 0.03, *p *= .018). For the baseline‐paired words, the simple effect of predictive duration was not significant, *F*(2, 22) = 0.45, *p* = .643, *η_p_* ^2^ = .04. For the distractor‐paired words, the simple effect of predictive duration was significant, *F*(2, 22) = 4.21, *p* = .028, *η_p_* ^2^ = .28. Corrected recognition rates in the MPD condition (0.14 ± 0.02) were marginally higher than in the SPD condition (0.09 ± 0.02, *p *= .073).

#### 3.2.2 Predictive distractors versus unpredictive distractors

**FIGURE 4:**
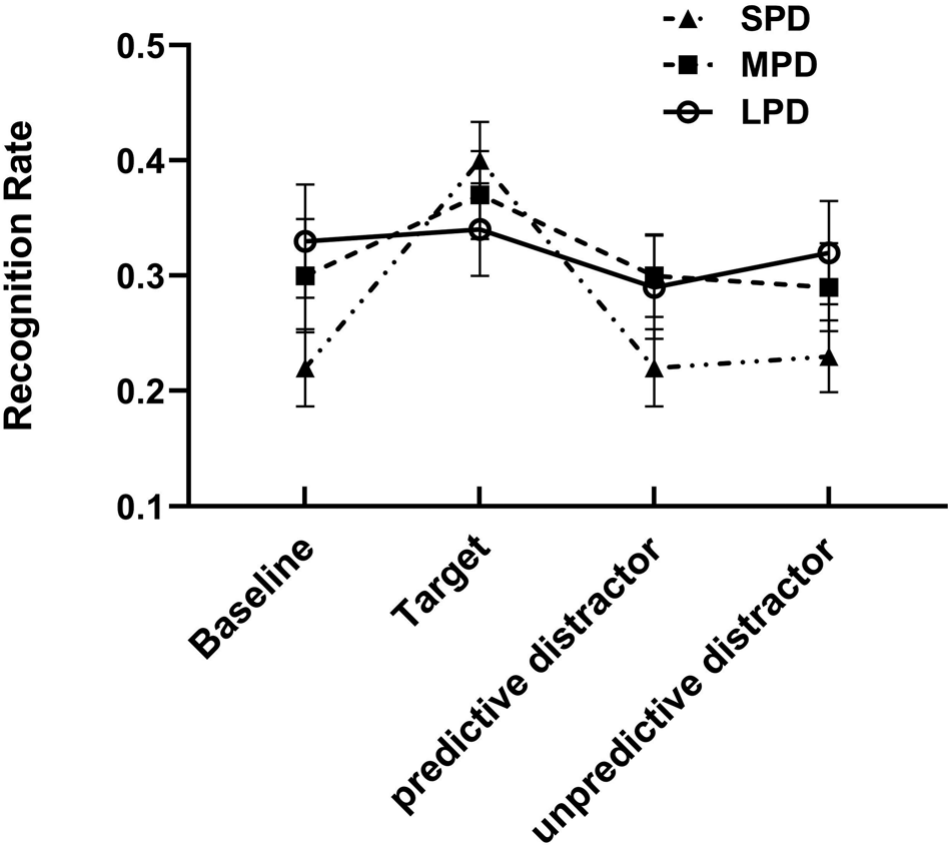
The corrected recognition rates for words paired with different detection stimuli types across the three predictive duration conditions. Error bars represent standard error of the mean.

In the current study, distractor digits can be categorized into two types: predictive and unpredictive. For instance, in the MPD conditions, the number 3, 2, 1 represents a predictive distractor digit, while the numbers 4–9 constitute unpredictive distractor digits. To explore the impact of predictability on recognition performance, a 3 (predictable duration: SPD, MPD and LPD) × 4 (detection stimuli type: target, baseline, predictive distractor and unpredictive distractor) repeated measures ANOVA on corrected recognition rates were conducted. The results (Figure 4) indicated that the main effect of predictable duration was not significant, *F*(1.52, 34.91) = 0.88, *p *= .398, *η_p_* ^2^ = .04. The main effect of detection stimuli type was significant, *F*(2.15, 49.47) = 20.91, *p* < .001, *η_p_* ^2^ = .48. Pairwise comparisons revealed that the corrected recognition rates for target‐paired words (0.22 ± 0.02) were significantly higher than those for baseline‐paired words (0.13 ± 0.02, *p* < .001), predictive distractor‐paired words (0.11 ± 0.01, *p* < .001) and unpredictive distractor‐paired words (0.12 ± 0.01, *p* < .001). No significant differences were found between corrected recognition rates for baseline‐paired words (0.13 ± 0.02), predictive distractor‐paired words (0.11 ± 0.01) and unpredictive distractor‐paired words (0.12 ± 0.01). Above repeated measures ANOVA also revealed a significant interaction between predictive duration and detection stimuli type, *F*(4.047, 93.089) = 6.02, *p* < .001, *η_p_* ^2^ = .21. Follow‐up simple effect analyses demonstrated a significant effect of detection stimuli type in the SPD condition, *F*(3, 21) = 17.51, *p* < .001, *η_p_* ^2^ = .71. Specifically, corrected recognition rates for target‐paired words (0.29 ± 0.03) were significantly higher than those for baseline‐paired words (0.11 ± 0.02, *p* < .001), predictive distractor‐paired words (0.09 ± 0.02, *p* < .001) and unpredictive distractor‐paired words (0.10 ± 0.02, *p* < .001). No significant differences were found between the corrected recognition rates for baseline‐paired words (0.11 ± 0.02), predictive distractor‐paired words (0.09 ± 0.02) and unpredictive distractor‐paired words (0.10 ± 0.02). In the MPD condition, the simple effect of detection stimuli type was significant, *F*(3, 21) = 4.33, *p* = .016, *η_p_* ^2^ = .38. Corrected recognition rates for target‐paired words (0.21 ± 0.03) were significantly higher than those for predictive distractor‐paired words (0.14 ± 0.02, *p* = .016) and unpredictive distractor‐paired words (0.13 ± 0.02, *p* = .007). No significant differences were found between the corrected recognition rates for baseline‐paired words (0.14 ± 0.03), predictive distractor‐paired words (0.14 ± 0.02) and unpredictive distractor‐paired words (0.13 ± 0.02). In the LPD condition, the simple effect of detection stimuli type was significant, *F*(3, 21) = 4.39, *p* = .015, *η_p_* ^2^ = .39. However, post hoc tests revealed that there were no significant differences in the corrected recognition rates between target‐paired words (0.15 ± 0.03), baseline‐paired words (0.13 ± 0.03), predictive distractor‐paired words (0.10 ± 0.02) and unpredictive distractor‐paired words (0.13 ± 0.02).

#### 3.2.3 Distal predictive nontarget digits versus proximal predictive nontarget digits

**FIGURE 5:**
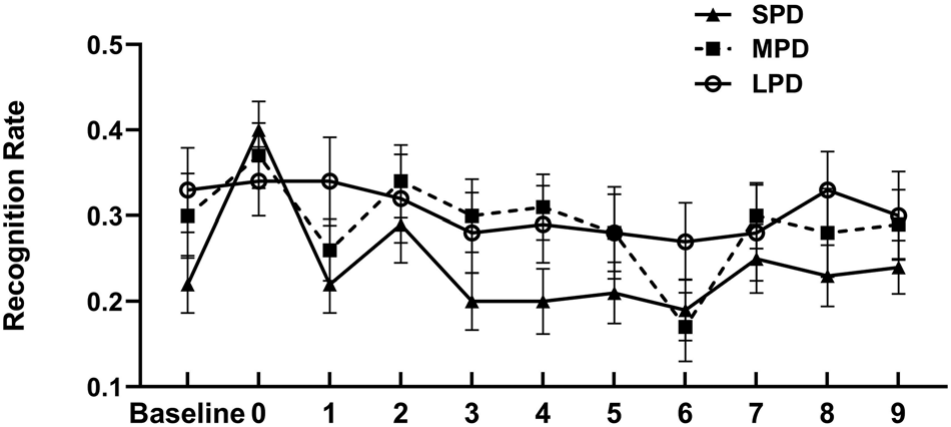
The corrected recognition rates for words paired with different detection digit across the three predictive duration conditions. Error bars represent standard error of the mean.

Predictive nontarget digits were categorized into two groups based on their distance from the target digit: distal predictive nontarget digits and proximal predictive nontarget digits. For example, in the MPD conditions, the number 3 represents a distal predictive nontarget digit, while the number 1 is a proximal predictive nontarget digit. In the LPD conditions, the number 7 serves as a distal predictive nontarget digit, while the number 1 serves as a proximal predictive nontarget digit. To further investigate the impact of temporal predictability on recognition performance, a one‐way ANOVA on corrected recognition rates was conducted separately for the MPD and LPD conditions. Results revealed that in the MPD condition, there was a significant difference in the corrected recognition rates of words associated with numbers 1, 2, and 3, *F*(2, 46) = 3.76, *p* = .031, *η_p_* ^2^ = .14 (Figure 5). Specifically, words coinciding with number 2 (0.18 ± 0.03) had a significantly higher corrected recognition rates than the words coinciding with number 1 (0.10 ± 0.02, *p* = .015). In the LPD condition, there was no significant difference in the recognition rates of words coinciding with numbers 1, 4, and 7, *F*(2, 46) = 1.63, *p* = .208, *η_p_* ^2^ = .07.

### 3.3 R/K response of recognition phase

#### 3.3.1 Target versus distractors

**FIGURE 6:**
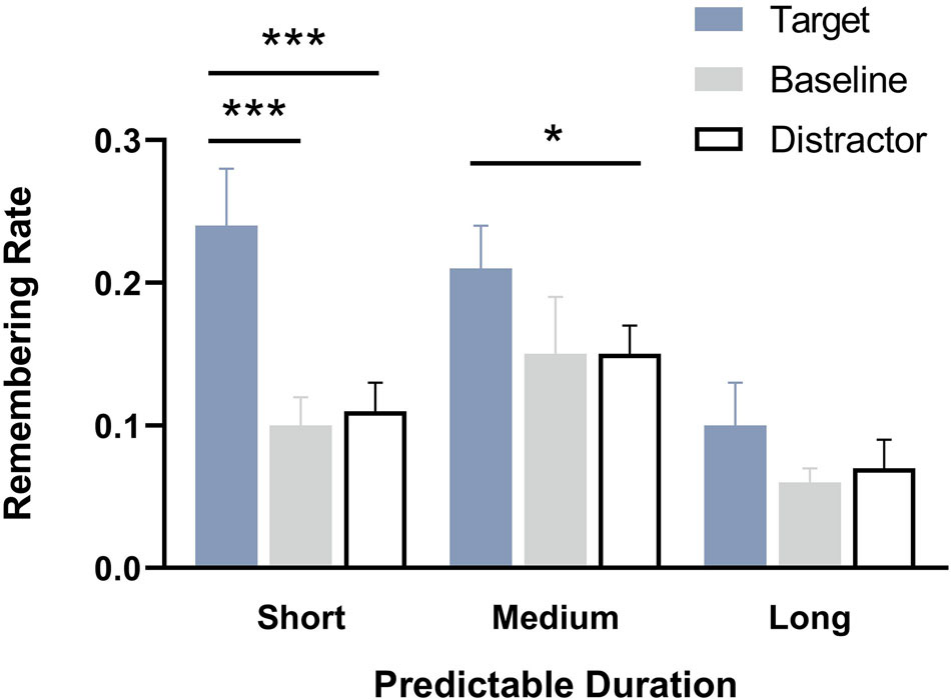
Remembering rates for words paired with different detection stimuli types across the three predictive duration conditions. Error bars represent standard error of the mean; *p < .05, **p < .01, ***p < .001.

For the R/K response, a 3 (predictive duration: SPD, MPD and LPD) × 3 (detection stimuli type: target, distractor and baseline) repeated measures ANOVA was conducted on remembering rates (Figure 6). Results indicated that the main effect of predictive duration was significant, *F*(2, 46) = 7.32, *p* = .002, *η_p_* ^2 ^= .24. Pairwise comparisons indicated that remembering rates in the LPD condition (0.09 ± 0.02) were significantly lower than that in the SPD condition (0.15 ± 0.02, *p* = .027) and MPD condition (0.16 ± 0.02, *p* = .008), with no significant difference between SPD condition and MPD condition (*p* = 1). The main effect of detection stimuli type was also significant, *F*(2, 46) = 33.26, *p* < .001, *η_p_* ^2^ = .59. Pairwise comparisons indicated that remembering rates for target‐paired words (0.19 ± 0.02) were significantly higher than baseline‐paired words (0.11 ± 0.02, *p* < .001) and distractor‐paired words (0.11 ± 0.01, *p* < .001), with no significant difference between baseline and distractor words (*p* = 1). The predictive duration × detection stimuli type interaction was also significant, *F*(2.28, 52.53) = 4.23, *p* = .003, *η_p_* ^2^ = .16. Simple effect analyses showed a significant effect of detection stimuli type in the SPD condition, *F*(2, 22) = 19.91, *p* < .001, *η_p_* ^2^ = .64. Remembering rates for target‐paired words (0.25 ± 0.03) were significantly higher than baseline‐paired words (0.10 ± 0.02, *p *< .001) and distractor‐paired words (0.10 ± 0.02, *p* < .001), while the differences of remembering rates between baseline‐paired words and distractor‐paired words were not significant (*p *= 1). Simple effects of detection stimuli type were also significant in the MPD condition, *F*(2, 22) = 5.34, *p* = .013, *η_p_* ^2^ = .33. Remembering rates for target‐paired words (0.20 ± 0.03) were significantly higher than those for distractor‐paired words (0.14 ± 0.02, *p *= .016). No significant differences were found between remembering rates for target‐paired words (0.20 ± 0.03) and baseline‐paired words (0.15 ± 0.03, *p* = .361), as well as between remembering rates for distractor‐paired words (0.14 ± 0.02) and baseline‐paired words (0.15 ± 0.03, *p* = 1). The simple effect of detection stimuli type in the LPD condition was not significant, *F*(2, 22) = 2.93, *p *= .075, *η_p_* ^2^ = .21.

We also conducted separate simple effect tests for different types of detection stimuli words at different predictive durations. The results showed that for the target‐paired words, the simple effect of predictive duration was significant, *F*(2, 22) = 9.01, *p* = .001, *η_p_* ^2^ = .45. Remembering rates in the SPD condition (0.25 ± 0.03) were significantly higher than in the LPD condition (0.12 ± 0.03, *p* < .001). Moreover, the remembering rates in the MPD condition (0.20 ± 0.03) were significantly higher than in the LPD condition (0.12 ± 0.03, *p *= .039). For the baseline‐paired words, the simple effect of predictive duration was not significant, *F*(2, 22) = 2.85, *p* = .079, *η_p_* ^2^ = .21. For the distractor‐paired words, the simple effect of predictive duration was significant, *F*(2, 22) = 6.08, *p* = .008, *η_p_* ^2^ = .36. Remembering rates in the MPD condition (0.14 ± 0.02) were marginally higher than in the SPD condition (0.10 ± 0.02, *p *= .015).

#### 3.3.2 Predictive distractors versus unpredictive distractors

**FIGURE 7:**
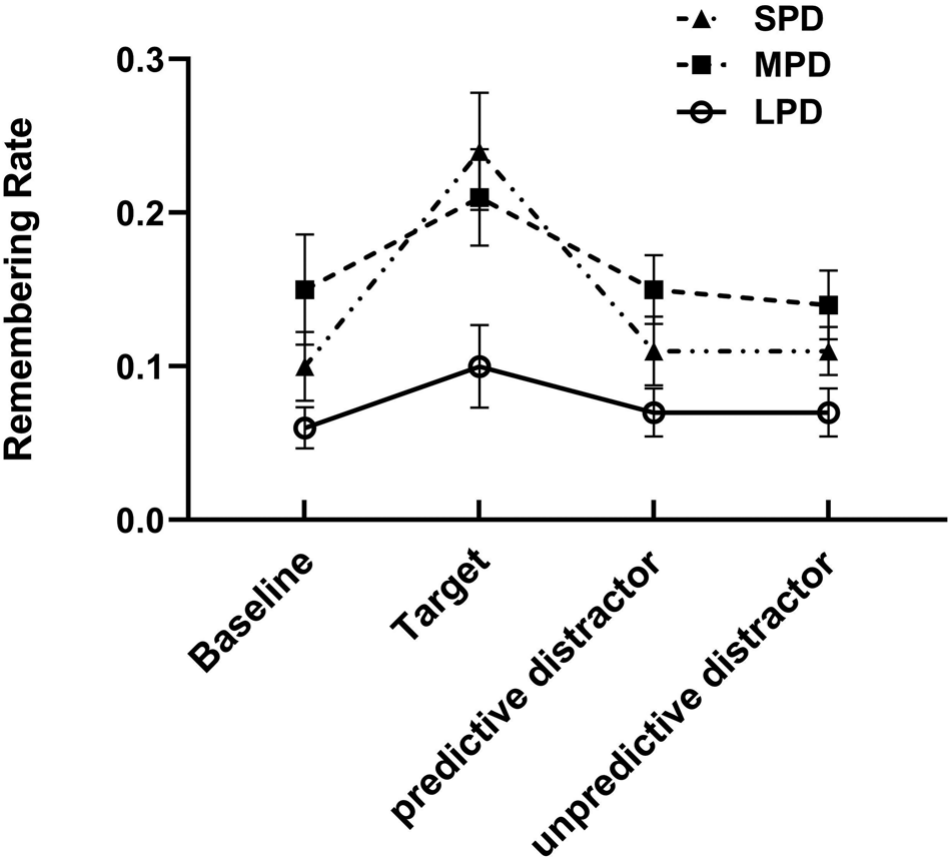
Remembering rates for words paired with different detection stimuli types across the three predictive duration conditions. Error bars represent standard error of the mean.

Similar to Section 3.2.2, 3 (predictable duration: SPD, MPD and LPD) × 4 (detection stimuli type: target, baseline, predictive distractor and unpredictive distractor) repeated measures ANOVA was performed on the remembering rates. The results (Figure 7) showed that the main effect of predictable duration was significant, *F*(2, 46) = 7.47, *p *= .002, *η_p_* ^2^ = .25. Pairwise comparisons indicated that remembering rates in the LPD condition (0.09 ± 0.01) were significantly lower than that in the SPD condition (0.14 ± 0.02, *p* = .040) and MPD condition (0.16 ± 0.02, *p* = .006), with no significant difference between SPD condition (0.14 ± 0.02) and MPD condition (0.16 ± 0.02, *p* = .721). The main effect of detection stimuli type was significant, *F*(2.33, 53.48) = 28.173, *p* < .001, *η_p_* ^2^ = .55. Pairwise comparisons revealed that the remembering rates for target‐paired words (0.19 ± 0.02) were significantly higher than those for baseline‐paired words (0.11 ± 0.02, *p* < .001), predictive distractor‐paired words (0.11 ± 0.02, *p* < .001) and unpredictive distractor‐paired words (0.11 ± 0.01, *p* < .001). No significant differences were found between remembering rates for baseline‐paired words (0.11 ± 0.02), predictive distractor‐paired words (0.11 ± 0.02) and unpredictive distractor‐paired words (0.11 ± 0.01). Above repeated measures ANOVA also revealed a significant interaction between predictive duration and detection stimuli type, *F*(3.13, 71.90) = 3.84, *p* = .012, *η_p_* ^2^ = .14. Follow‐up simple effect analyses demonstrated a significant effect of detection stimuli type in the SPD condition, *F*(3, 21) = 13.44, *p* < .001, *η_p_* ^2^ = .66. Specifically, remembering rates for target‐paired words (0.25 ± 0.03) were significantly higher than those for baseline‐paired words (0.10 ± 0.02, *p* < .001), predictive distractor‐paired words (0.10 ± 0.02, *p* < .001) and unpredictive distractor‐paired words (0.10 ± 0.02, *p* < .001). No significant differences were found between the remembering rates for baseline‐paired words (0.10 ± 0.02), predictive distractor‐paired words (0.10 ± 0.02) and unpredictive distractor‐paired words (0.10 ± 0.02). In the MPD condition, the simple effect of detection stimuli type was significant, *F*(3, 21) = 3.49, *p* = .034, *η_p_* ^2^ = .33. Remembering rates for target‐paired words (0.20 ± 0.03) were significantly higher than those for predictive distractor‐paired words (0.14 ± 0.02, *p* = .036) and unpredictive distractor‐paired words (0.14 ± 0.02, *p* = .037). No significant differences were found between the remembering rates for baseline‐paired words (0.15 ± 0.03), predictive distractor‐paired words (0.14 ± 0.02) and unpredictive distractor‐paired words (0.14 ± 0.02). In the LPD condition, the simple effect of detection stimuli type was not significant, *F*(3, 21) = 2.49, *p* = .089, *η_p_* ^2^ = .26.

#### 3.3.3 Distal predictive nontarget digits versus proximal predictive nontarget digits

**FIGURE 8:**
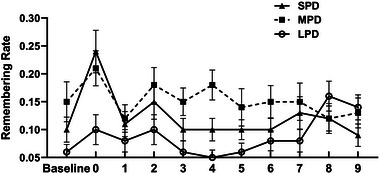
The remembering rates for words paired with different detection digit across the three predictive duration conditions. Error bars represent standard error of the mean.

Similar to Section 3.2.3, a one‐way ANOVA on remembering rates was conducted separately for the MPD and LPD conditions to further investigate the impact of predictability on memory performance. Results revealed that (Figure 8) in the MPD condition, there was a marginally significant difference in the remembering rates of words associated with numbers 1, 2, and 3, *F*(2, 46) = 2.71, *p* = .077, *ηp* ^2^ = .11. Specifically, words coinciding with number 2 (0.17 ± 0.03) had a marginally higher remembering rates than words coinciding with number 1 (0.12 ± 0.02, *p* = .062). In the LPD condition, there was no significant difference in the remembering rates of words associated with numbers 1, 4, and 7, *F*(2, 46) = 2.23, *p* = .119, *ηp* ^2^ = .09.

#### 3.3.4 Knowing rates of memory task

**FIGURE 9:**
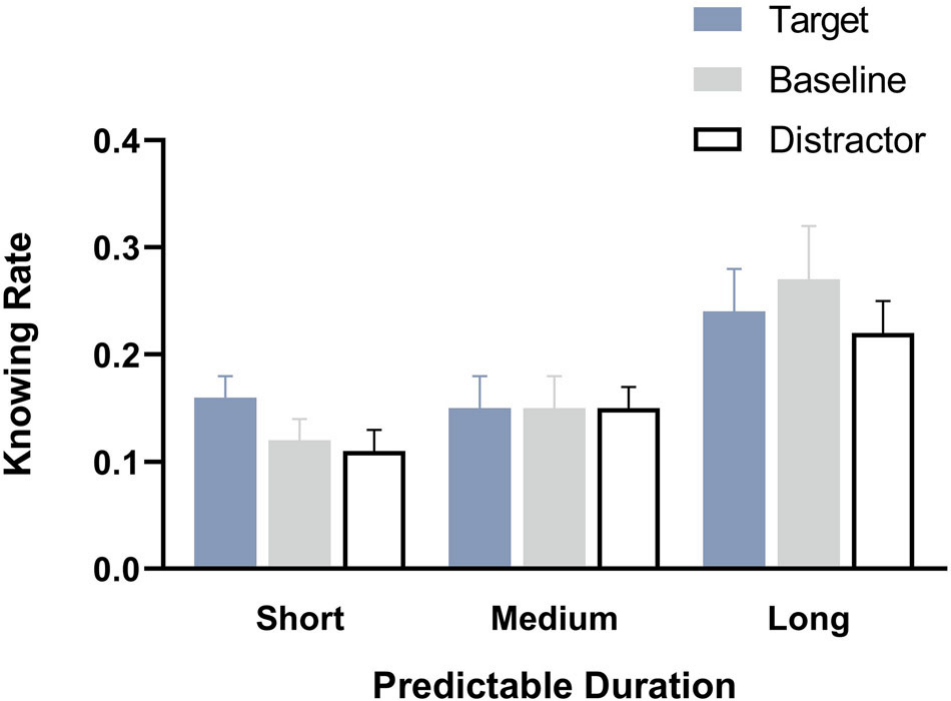
Knowing rates for words paired with different detection stimuli types across the three predictive duration conditions. Error bars represent standard error of the mean; *p < .05, **p < .01, ***p < .001.

A 3 (predictive duration: SPD, MPD and LPD) × 3 (detection stimuli type: target, distractor and baseline) repeated measures ANOVA was conducted on knowing rates (Figure 9). Results indicated a significant main effect of predictive duration, *F*(1.43, 32.97) = 6.50, *p* = .009, *η_p_* ^2^ = .22. Post hoc tests revealed that knowing rates in the LPD condition (0.22 ± 0.03) were significantly higher than those in the SPD condition (0.13 ± 0.01, *p* = .025) and were marginally significantly higher than those in the MPD condition (0.15 ± 0.02, *p* = .072). However, the differences between knowing rates in the SPD condition (0.13 ± 0.01) and the MPD condition (0.15 ± 0.02) were not significant (*p* = .616); The main effect of detection stimuli type was not significant, *F*(1.41, 32.25) = 1.347, *p *= .266, *η_p_* ^2^ = .06; The predictive duration × detection stimuli type interaction was also not significant, *F*(4, 92) = 1.09, *p *= .367, *η_p_* ^2 ^= .05. Given that recognition rates predominantly stem from remembering rates, a detailed analysis akin to Sections 3.2.2 and 3.2.3 for knowing rates is not provided.

## 4 DISCUSSION

Sisk and Jiang's study demonstrated that predictive target stimuli can also trigger the ABE (Sisk & Jiang, 2020). However, their study employed a fixed predictive duration (3 s), preventing further investigation into the impact of different predictive durations on the ABE. Using the encoding‐recognition paradigm and the remembering/knowing paradigm, and setting target stimuli with different predictive duration in target detection tasks. The current study aimed to explore the influence of varying predictive durations on the ABE, thereby providing a more nuanced understanding of the impact of temporal predictability on the ABE.

### 4.1 Target versus nontarget: the modulate effect of temporal predictability on ABE

The repeated measures ANOVA on corrected recognition rates and remembering rates consistently revealed that, in both the SPD and MPD conditions, the corrected recognition rates and remembering rates for target‐paired words were significantly higher than those for distractor‐paired words. While in the LPD condition, no significant differences were observed between the corrected recognition/remembering rates of target‐paired and distractor‐paired words (Figures 3 and 6). That is to say, the superiority of remembering target‐paired words over distractor‐paired words was evident only in the SPD and MPD conditions. According to the operational definition of ABE (Sisk & Jiang, 2020), these results suggest that, in the current study, ABE is observed exclusively in the SPD and MPD conditions, which is consistent with both previous study (Sisk & Jiang, 2020) and our hypothesis.

Because the ABE is thought to arise from a transient increase in attentional capacity (perceptual enhancement) at the time of an acute behavior‐related event occurred (Sisk & Jiang, 2020). The current study's observation that ABE is evident only in the SPD and MPD conditions, but not in the LPD conditions, may be attributed to the target stimuli in SPD and MPD conditions possessing sufficient acuteness to elicit a robust perceptual enhancement. In contrast, the LPD condition extended the anticipation and preparation for the target's appearance, resulting in diminished acuteness and a weakened perceptual enhancement of temporal selective attention.

In addition, as depicted in Figures 3 and 6, the corrected recognition/remembering rates of target‐paired words demonstrated a declining trend with the prolonged predictive duration, while the corrected recognition/remembering of baseline‐paired and distractor‐paired words exhibited an increasing trend with the extension of predictive duration. Accordingly, we can predict that individuals were able to flexibly adjust their attentional allocation strategies according to the task urgency predictability. With the increase in predictive duration, in addition to target stimuli, subjects also allocate part of their attentional resources to nontarget stimuli, which weakens the attentional resource difference between the target stimulus and distractor stimulus, thereby preventing the ABE from manifesting.

### 4.2 Predictive distractors versus unpredictive distractors

In the current study, distractor digits can be further categorized into predictive distractor digits and unpredictive distractor digits. For example, in the MPD conditions, the digits 3, 2, and 1 represent predictive distractor digits, while the digits 4–9 constitute unpredictive distractor digits. To explore the impact of predictability on memory performance, a 3 (predictable duration: SPD, MPD, and LPD) × 4 (detection stimuli type: target, baseline, predictive distractor, and unpredictive distractor) repeated measures ANOVA on corrected recognition rates and remembering rates were conducted. The results (Figures 4 and 7) indicated that, in any of the three predictable conditions, no significant differences were found in corrected recognition and remembering rates between words paired with predictive distractors and words paired with unpredictive distractors. This finding is inconsistent with Sisk and Jiang's results, as they found a better memory for scenes encoded with the predictive digits 3−1 compared to the unpredictive digits 7−9. One possible explanation for this discrepancy is that the memory materials used in the two experiments were different. In addition, it is worth noting that the memory benefits of predictive distractors compared to unpredictive distractors only occurred in experiment 1 but not in experiments 2 and 3 of Sisk and Jiang's study. While the only difference between experiment 1 and experiments 2 and 3 was the addition of a 0‐distractor condition in the later experiments. This suggests that the memory benefits of predictive distractors compare to the unpredictive may be not stable, it was influenced by a variety of factors, such as experimental conditions and remember materials. The underlying mechanism of these memory benefits of predictive distractors warrants further exploration in future studies.

### 4.3 Distal predictive nontarget digits versus proximal predictive nontarget digits

In the current study, the countdown number strings (SPD: 1‐0, MPD: 3‐2‐1‐0, LPD: 7‐6‐5‐4‐3‐2‐1‐0) were always presented in a fixed order. Based on the distance from the target digit, predictive nontarget digits can be further categorized into distal predictive nontarget digits and proximal predictive nontarget digits. For instance, in the MPD conditions, the digit 3 represents a distal predictive nontarget digit, while the digit 1 is a proximal predictive nontarget digit. In the LPD conditions, the digit 7 serves as a distal predictive nontarget digit, while the number 1 serves as a proximal predictive nontarget digit. Because the distance from the target digit was different, these predictive nontarget digits may serve different roles. For instance, proximal predictive nontarget digits may indicate of the onset of the sequence, and distal predictive nontarget digits may indicate the upcoming target. To further investigate whether word memory differs for these predictive nontarget digits, a one‐way ANOVA on corrected recognition rates and remembering rates was conducted separately for the MPD and LPD conditions. Results revealed that, for both corrected recognition rates and remembering rates, in the MPD condition, words coinciding with number 2 indicated a memory advantages compared to words coinciding with number 1 (Figures 5 and 8). These findings were consistent with the results of Sisk and Jiang's study, which showed a small but significant memory advantage for scenes coinciding with the center digit 2, but not the bilateral digit 1 or 3.

The above findings confirm that predictive nontarget digits with different distance from the target digit indeed serve different roles. When the distal predictive nontarget digits (e.g. digit 3 in MPD condition) occur, participants may not have sufficient time to allocate attention to the background words, but they serve as a warning cue, enabling more effective resource allocation when the middle digits (e.g., digit 2 in the MPD condition) are present. Although the same task management applies to proximal digits (e.g., digit 1 in the MPD condition), memory performance for words coinciding with digit 1 in the MPD condition did not exhibit the same memory advantage. This difference may result from response anticipation. Previous research has found that, when the study phase involved a regular alternation between blocks of five target trials and blocks of five distractor trials, the recognition of distractor‐paired words decreased linearly as the first target trial approached, due to the negative effects of response anticipation (Saraulli et al., 2023). This anticipatory effect on recognition memory could result in poorer recognition/remembering of words appearing just before the target digit 1 in MPD condition. The warning effect of distal predictive digits and the anticipatory effect of proximal digits commonly resulted in the intermediate position memory advantages of middle digits.

**FIGURE 10:**
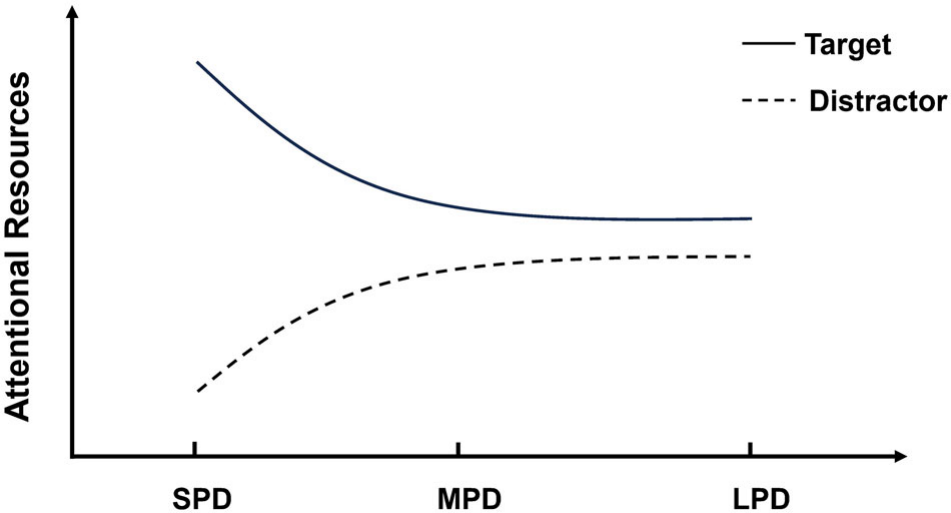
Task acuteness affects individuals’ attentional allocation to different types of detection stimuli.

Interestingly, such memory advantages of words coinciding with center number compared to words coinciding with bilateral number, that is, intermediate position memory advantages, were not observed in the LPD condition of the current study (Figures 5 and 8). In addition, in the LPD condition, we did not observe the ABE, indicating that the difference between the memory performance of words coinciding with target digit and words coinciding with the distractor nontarget digit was insignificant. Previous research has find that temporal predictability alters the pattern of mind wandering (Seli et al., 2018) and allows for better cognitive control in dual‐task conditions (Whitehead & Egner, 2018). Because the longer predictive duration means higher predictability in LPD condition, combined with the current results and the aforementioned previous research, we can predict that longer predictability in a dual‐task scenario allows participants have sufficient time to flexibly allocate their attention resources to both target stimuli and the background words, thereby improving performance on both tasks (Figure 10).

## 5 LIMITATIONS AND FUTURE DIRECTIONS

Our research was not without limitations. Firstly, the number of predictive distractors and unpredictive distractors was unequal in our study, which may have hindered comparisons with previous studies. Secondly, the conclusions of the current study are based on reasoning derived from behavioral data. Follow‐up studies can provide support for these inferences through neuroimaging techniques such as Event‐Related Potentials (ERPs) and functional Magnetic Resonance Imaging (fMRI), which will help us better understand the neural mechanisms of the ABE.

## 6 CONCLUSIONS

Regarding the connection between event predictability and ABE, we propose a relationship as depicted in Figure 10. Predictability may alter the task demands, allowing participants to more effectively allocate attentional resources to the two tasks at hand. Specifically, under conditions of short predictability (e.g., SPD condition), individuals tend to preferentially allocate more attentional resources to the target stimulus, which requires acute processing, resulting in the words coinciding with the target stimulus being more deeply processed and thus showing a memory advantage (ABE). In contrast, under conditions of long predictability (e.g., LPD condition), individuals have sufficient time to allocate attentional resources to a wider range of stimuli. In addition to target stimuli, subjects also allocate part of their attentional resources to nontarget stimuli (e.g., distractor stimuli). The attentional resources allocated to nontarget stimuli increase with the extension of duration of the predictive interval, weakening the difference between the target stimulus and distraction stimulus, thereby preventing the ABE from appearing.

## AUTHOR CONTRIBUTIONS

**Jianan Pan**: Conceptualization; methodology; software; formal analysis; writing—original draft. **Chao Fu**: Conceptualization; writing—review & editing. **Ping Su**: Revision; polish. **Qian Guo**: Writing—review & editing. **Xinglin Li**: Critical reading; comprehensive check. **Chun Zheng**: Revision; polish; critical reading; comprehensive check. **Xueqin Ma**: Critical reading; comprehensive check. **Tingjun Yong**: Critical reading; comprehensive check.

## CONFLICT OF INTEREST STATEMENT

The authors declared no conflicts of interest with respect to the authorship or the publication of this article.

### INFORMED CONSENT STATEMENT

Written informed consent was obtained from all the participants prior to the enrollment of this study.

### PEER REVIEW

The peer review history for this article is available at https://publons.com/publon/10.1002/brb3.3653.

## Data Availability

The data that support the findings of this study are available from the corresponding author upon reasonable request.
